# Balancing Urgency and Ethics in the Kyasanur Forest Disease Investigation in Shivamogga: The Outbreak Dilemma

**DOI:** 10.3389/ijph.2025.1608861

**Published:** 2025-08-22

**Authors:** Srividya K. Vedachalam, Sushma Choudhary

**Affiliations:** 1 National Centre for Disease Control, New Delhi, India; 2 South Asia Field Epidemiology and Technology Network, New Delhi, India

**Keywords:** ethical review, social responsibility, disease outbreaks, informed consent, confidentiality

We appreciate the opportunity to respond to the comments raised regarding our manuscript titled “Kyasanur Forest Disease: An epidemiological investigation and case-control study in Shivamogga, Karnataka, India-2022 [1].” We thank the author of the letter for their interest in our work [2]. However, we respectfully disagree with several of the points raised and would like to clarify our position below.

The World Health Organization’s document, *“Guidance for Managing Ethical Issues in Infectious Disease Outbreaks”* (2016), referred to by the author of the letter, specifically Chapter 8 (pp. 30–34) titled *“Research during infectious disease outbreaks,”* emphasizes the critical role of research—including epidemiological, social science, and implementation research—during outbreaks to help reduce morbidity and mortality [3]. It acknowledges that the ability to obtain a formal ethical review may be constrained during public health emergencies due to time pressures. However, it stresses that such research must remain scientifically valid and uphold core ethical principles. The document outlines several steps through which researchers can ensure transparency and ethical conduct, even in the absence of a formal review. In line with this guidance, we wish to enumerate these recommended steps and demonstrate how our team adhered to each of them, ensuring ethical rigor and transparency throughout the investigation process.

1. Involvement of local researchers during the design, implementation, analysis and reporting stage: Our author list includes local state and district officials who participated at each step of the outbreak response. This ensured that the investigation was aligned with the local authorities’ goals of outbreak control along with capacity building of local health staff.
2. Maintaining patient confidentiality: All data was obtained after informed consent. This was an observational investigation. No interventions were carried out other than that required for the outbreak response. Only aggregate data was shared for publication, no individual level data was shared.
3. Ensuring that research does not drain critical health-related resources: The investigation was conducted as part of outbreak response and field teams and health staff were involved only for the duration of the outbreak response.
4. Rapid data sharing: All data regarding the outbreak collected during field investigation was shared with the local state and national authorities daily to respond to the evolving evidence on the field. All the communication is documented in emails and archived. As stated above, this investigation was part of an outbreak response and not a stand-alone research project where results are shared with stakeholders only during publication.
5. Assuring equitable access to benefits of research: As a result of this investigation a national consultative meeting was organized with the state health authorities to discuss the way forward for the KFD vaccine. Thus, the benefits of the research were channeled back to the community from where the research was carried out.
6. Providing ethics review in time-sensitive circumstances:

Currently, there is no mechanism within our state or national public health systems to conduct an accelerated ethics review during outbreak situations. As a result, we were unable to obtain such a review, as suggested by the author of the letter. Nevertheless, we made every effort to maintain ethical integrity and transparency throughout the investigation, as outlined in the preceding points. Moreover, conducting an analytical investigation as part of outbreak response to identify risk factors and aid in control measures is an integral part of outbreak investigation and is not considered generating generalizable knowledge. Therefore, we respectfully disagree with the assertion that “the investigation raises ethical questions about transparency and accountability.” We believe our approach aligned with the WHO guidance for outbreak research in emergency settings, particularly in upholding scientific rigor and ethical conduct in the absence of formal review processes.

The issue of ethics preparedness during outbreaks, as raised in the query, is highly pertinent—particularly in light of the experiences during the Ebola and COVID-19 pandemics. A workshop convened in May 2018 by the WHO Global Health Ethics Team and the African Coalition for Epidemic Research, Response, and Training (WHO ALERRT) recommended the development of formal national standard operating procedures (SOPs) for ethical review during emergency responses [4]. However, until such frameworks are implemented at the ground level, it remains essential for public health professionals engaged in outbreak response to share their findings with the scientific community. The lack of a formal ethical review process for outbreak investigations often poses a barrier in sharing critical findings with the public health community through scientific publications. As a result, valuable insights generated from real public health challenges faced during outbreak responses remain unpublished, depriving the global scientific community of evidence that can improve future preparedness and response strategies. In this context, we are thankful to journals like the *International Journal of Public Health (IJPH)* that recognize the urgency of outbreak-related research and support the dissemination of such findings—even in the absence of a formal ethics review process—while upholding scientific rigor and public health relevance.
